# In Flight Performance of the Far Ultraviolet Instrument (FUV) on ICON

**DOI:** 10.1007/s11214-023-00969-9

**Published:** 2023-03-28

**Authors:** H. U. Frey, S. B. Mende, R. R. Meier, U. Kamaci, J. M. Urco, F. Kamalabadi, S. L. England, T. J. Immel

**Affiliations:** 1 Space Sciences Laboratory, University of California, Berkeley, CA USA; 2 Department of Physics and Astronomy, George Mason University, Fairfax, VA USA; 3 Department of Electrical and Computer Engineering, University of Illinois at Urbana-Champaign, Urbana, IL USA; 4 Department of Aerospace and Ocean Engineering, Virginia Polytechnic Institute and State University, Blacksburg, VA USA; 5 Present Address: Leibniz-Institute for Atmospheric Physics, Kühlungsborn, Germany

**Keywords:** Ionosphere, Airglow, NASA ICON mission, FUV instrument

## Abstract

The NASA Ionospheric Connection Explorer (ICON) was launched in October 2019 and has been observing the upper atmosphere and ionosphere to understand the sources of their strong variability, to understand the energy and momentum transfer, and to determine how the solar wind and magnetospheric effects modify the internally-driven atmosphere-space system. The Far Ultraviolet Instrument (FUV) supports these goals by observing the ultraviolet airglow in day and night, determining the atmospheric and ionospheric composition and density distribution. Based on the combination of ground calibration and flight data, this paper describes how major instrument parameters have been verified or refined since launch, how science data are collected, and how the instrument has performed over the first 3 years of the science mission. It also provides a brief summary of science results obtained so far.

## Introduction

The interaction between neutrals and ions mediated by electric fields and neutral winds and forcing from the lower atmosphere determine the coupling across atmospheric regions. Determining the details of this interaction requires a remote sensing space mission as long-term continuous in situ measurements at the altitudes of interest (90–300 km) are impossible. NASA selected the Ionospheric Connection Explorer (ICON) mission to investigate the details of that coupling by making continuous observations of the neutral atmospheric drivers and determine the ionospheric responses. ICON was launched in October 2019 and has three science objectives, to understand: 1. the sources of strong ionospheric variability; 2. the transfer of energy and momentum from our atmosphere into space; and 3. how solar wind and magnetospheric effects modify the internally-driven atmosphere-space system (Immel et al. 2018).

The ICON satellite flies in a $578 \times 607~\text{km}$ orbit at $27.0^{\circ}$ inclination. The remote sensing instruments are nominally pointed perpendicular to the velocity vector and normally look towards the north in the usual science data taking orientation of the spacecraft. The satellite carries four science instruments and one of them, the Far Ultraviolet Instrument (FUV), supports these science objectives by observing the upper atmospheric airglow at 135.6 nm and 157 nm (Mende et al. 2017). Observations in the far ultraviolet from space have the advantage over observations in the visible part of the spectrum because the atmosphere is opaque below about 100 km altitude due to absorption by molecular oxygen. A downward viewing ultraviolet (UV) instrument thus does not suffer from contamination by, for instance, Rayleigh-scattered sunlight or moonlight from clouds or the ground.

On the dayside, UV airglow is generated by photoelectron impact on neutral species (primarily O and N_2_), putting these atoms/molecules into excited quantum states that then relax through photon emission at specific UV wavelengths. The intensity of those emissions can be used to determine the composition ratio of these major atmospheric species as this is one of the major drivers of the system.

Oxygen atoms that were ionized on the dayside last into the dark nightside due to the relatively long life time of the $\text{O}^{+}$ ion at higher altitudes, leading to a delayed recombination and emission of 135.6 nm photons. The 135.6 nm emission intensity is proportional to the recombination rate and thus reflects the product of $\text{O}^{+}$ ion density and of the electron density. Consequently, the intensity of the 135.6 nm emission can be used to obtain the $\text{O}^{+}$ ion density of the nighttime ionosphere (Mende et al. 2017).

The FUV instrument supports the ICON science goals (Immel et al. 2018) by providing the information necessary to determine the daytime thermospheric density profiles of the neutral species O and N_2_. Furthermore, it provides radiance measurements that determine the nighttime $\text{O}^{+}$ ion density. Details of the retrieval algorithms for the thermospheric composition are described in Stephan et al. (2018) with updates to the disk algorithm for column O/N_2_ retrieval by Meier (2021). The retrieval algorithm for the nighttime ionospheric density is given in Kamalabadi et al. (2018).

## FUV Instrument Design and Measurement Principle

The FUV instrument, its design, characteristics, and ground calibration were described in great detail in Mende et al. (2017). Here we will only give a brief summary.

The ICON FUV instrument is a 2-channel grating-based Spectrographic Imager (Mende 2016) and it detects the oxygen atom 135.6 nm emission (short-wave or SW-channel) and a portion of the nitrogen molecule Lyman-Birge-Hopfield (LBH) emission (long-wave or LW-channel) around 157 nm (Mende et al. 2017). The FUV instrument has an $18^{\circ}\times24^{\circ}$ ($\text{horizontal}\times \text{vertical}$) field of view (FOV) and is pointed $20^{\circ}$ downward from the local horizontal plane. The observations thus cover an altitude range from about 530 km tangent height down to the sub-limb region $58^{\circ}$ off nadir. The 256 nominal science pixels allow for an altitude resolution of 4 km at the limb (Mende et al. 2017). A movable turret was added as part of the FUV instrument that can point the instrument FOV in $10^{\circ}$ steps to up to $\pm30^{\circ}$ from the nominal view direction which is perpendicular to the satellite velocity vector. This allows to rotate the azimuth of the FUV view axis in order to point along the local magnetic meridian.

One of the dominant ultraviolet emissions from oxygen atoms is the doublet (^3^P_2_–^5^S_2_) and (^3^P_1_–^5^S_2_) at 135.56 nm and 135.85 nm, which generally is designated as 135.6 nm emission (Meier 1991). One of the dominant ultraviolet emissions from nitrogen molecules is the Lyman-Birge-Hopfield band ($\text{a}^{1}\Pi _{\mathrm{g}}$–$\text{X}^{1}\Sigma_{\mathrm{g}}^{+}$ transition) with many emission lines between 120–280 nm (Bishop and Feldman 2003). Previous space missions, for instance the Thermosphere Ionosphere Mesosphere Energetics and Dynamics (TIMED) mission with the Global Ultraviolet Imager (GUVI, Paxton et al. 1999) observed the 135.6 nm (OI) emission and a portion of the N_2_ LBH bands to determine the altitude profiles of the number densities and temperatures of these emitting species. From those observations it determined the composition of the ionosphere on the dayside (Christensen et al. 2003). Similar measurements are now done by the Global-Scale Observations of the Limb and Disk (GOLD) mission (McClintock et al. 2020).

FUV uses microchannel plates (MCP) in front of the CCD. These MCPs accelerate and multiply photoelectrons that are generated at the front photocathode by the incoming photons. The electron cloud generates a strong light flash on the phosphor screen at the MCP output and the CCD generates an analog signal proportional to the amount of light impinging on the CCD pixels. The CCD signal is digitized with an analog to digital (A/D) converter with a 14-bit readout. Both the A/D gain and the digital number representing the dark signal are parameters that are adjustable in flight. All science data and calibration outputs are discussed here in terms of the digital units generated by the A/D converter. These units are often referred to as “A/D counts” or simply “counts” but they should not be confused with photoelectron counts.

## Altitude Profile Data Collection

The ICON FUV is a spectrographic imager that simultaneously generates two-dimensional images of the observed scene in the two independent wavelength regions. In order to obtain the required signal to fulfill the instrument requirements (see Mende et al. 2017), an integration time of 12 seconds per record is used. At the ICON satellite altitude of $\sim600~\text{km}$ the spacecraft moves $\sim90~\text{km}$ horizontally during that time and integrations over such times are smeared because of the spacecraft motion. In order to minimize smearing, a co-adding process was developed for which the instrument takes 100 exposures of 120 msec each. Each exposure is collapsed horizontally into 6 stripes representing $24^{\circ}$ vertical size and $3^{\circ}$ in horizontal width. The 100 exposures are then summed in the direction of spacecraft motion without smearing in the vertical direction (for details see Mende et al. 2017).

**Fig. 1:**
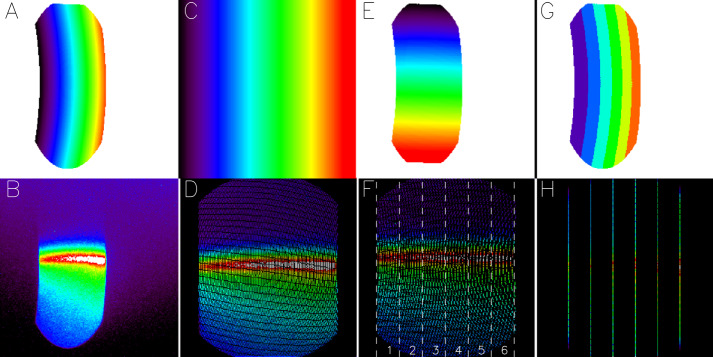
Demonstration of the ICON FUV data collection process for the generation of altitude profiles. Panel **B** shows a two-dimensional image of the airglow at the Earth limb. Geometric distortions by the optical system (Panel **A**) are corrected to finally get the airglow altitude profiles shown in panel **H**. The individual panels are explained in the text

All optical systems suffer from some level of geometric distortion that will bend a straight line object into a curved line in the image. In order for the co-adding technique to work properly, such geometric distortions have to be accommodated before the individual frames are co-added in memory. Before integration of the FUV instrument into the ICON satellite, a dedicated calibration campaign was performed (Frey et al. 2017) which determined not only the geometric imaging properties of the system but also the passbands, field of view, out of band contributions from scattering, and the overall quantitative sensitivity of the system (Mende et al. 2017). The results of the calibration campaign were used to determine geometric distortion correction tables that were loaded to the instrument and are applied on board. The full process of altitude profile data collection is shown in Fig. 1 and described in the following paragraphs.

As noted above, the overall field of view of the instrument is $24^{\circ}$ in the vertical direction and $18^{\circ}$ in the horizontal direction, restricted by the size and shape of the grating. Panel A of Fig. 1 shows the geometric distortion that the optical system applies to the incoming light. It demonstrates how the distortion affects the vertical and horizontal directions differently, as the vertical $24^{\circ}$ are displayed in 250 pixels while the horizontal $18^{\circ}$ are imaged in only 100 pixels. Panel B shows a true two-dimensional SW-channel image of the airglow at the dayside limb. Panel C shows the geometric distortion correction that transforms the image in Panel B into the straightened out image shown in Panel D. Now distances in the vertical and horizontal directions are equal. There are also angular corrections (Panel E) that need to be applied and even though in this particular example this correction is small, it leads to the final corrected frame shown in Panel F. The vertical dashed lines mark $3^{\circ}$ wide horizontal regions. The numbers refer to the stripe numbers that will later be used in this paper. The straightened frames of Panel F are then co-added in memory.

The processing works with look-up tables of pixel addresses and Panel G shows in six different colors which of the pixels in the original image are collapsed in the horizontal direction. The resulting stripes are shown in Panel H. Instead of $256\times256$ pixel 32-bit images ICON only transmits $6\times256$ pixel 32-bit profiles with real information (plus some auxiliary data). This compression method cuts the transmitted data volume by a factor of about 40 for each channel. Because of the horizontal binning of the $3^{\circ}$ wide regions ($\sim 130~\text{km}$ at the 150 km altitude limb in 2500 km distance) there is no need for a horizontal offset before co-adding of altitude profiles (see below for the treatment of mapped images).

**Fig. 2:**
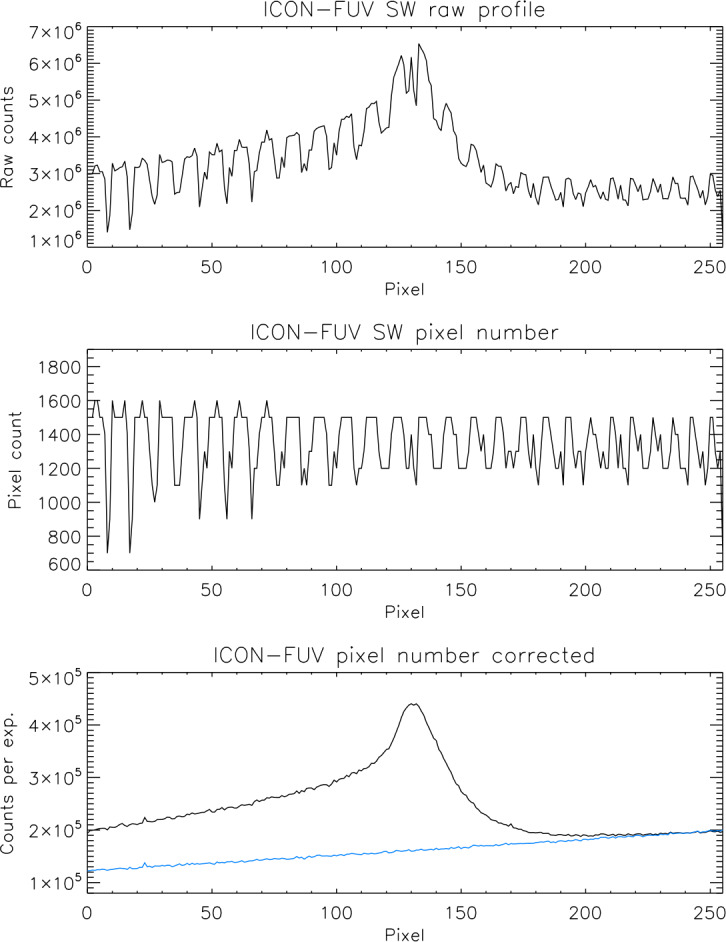
Demonstration of the science altitude profile generation. The top panel shows the raw profile as it is down linked from the spacecraft, the middle panel shows the number of raw pixels added into each vertical science pixel, and the bottom shows the pixel number corrected altitude profile. The blue line shows the CCD count background that is subtracted before the quantitative calibration. The pixel numbers correspond to the vertical view directions from $-32^{\circ}$ to $-8^{\circ}$ angle with respect to the spacecraft horizontal plane

Panel F shows that the correction process leaves empty pixels or “holes” without content as the raw image is stretched out. One could consider some interpolation scheme to fill these holes but that would either generate additional signal, or the content of original pixels would have to be spread out over several pixels in some weighted manner. As ICON FUV should report the true signal is was decided not to implement an interpolation but rather add all the original signal into the final $0.09375^{\circ}\times3^{\circ}$ science pixels. This leads to different numbers of raw pixels that are added in neighboring final science pixels and is demonstrated in Fig. 2.

The top panel shows a raw altitude profile that is generated during the co-adding process and sent down to the ground. The rugged appearance is the result of the varying number of raw pixels that go into vertical science pixels while maintaining their position in altitude. The middle panel shows the number of raw pixels that were added into each science pixel which for the SW-channel here is on average 14 pixels from all of the 100 raw frames. After dividing the raw profiles by the number of active pixels in each science pixel we get the pixel number corrected profile in raw CCD counts per 12 seconds integration as shown in the bottom panel.

## Nighttime 2D Image Data Collection

The nightside equatorial ionosphere can be highly structured due to neutral wind and electric field forces and the resulting equatorial anomaly (see e.g. Schunk and Nagy 2009) can show plasma density depletions or bubbles (Henderson et al. 2005). Such density modulations can be seen in the nightglow (Hicks and Chubb 1970). The depletions create longitudinally narrow reductions in the nightglow emission at 135.6 nm that are highly extended along the local magnetic field. These bubbles are one of the main science targets for the nighttime observations by FUV.

As the spacecraft moves $\sim90~\text{km}$ in 12 s, the signal from ionospheric modulations would be smeared during a continuous integration. In order to capture fine structures with FUV, we decided to use the time delay integration (TDI) technique (Mende et al. 2000) for nighttime imaging. Individual frames are collected for 120 ms, and 100 frames are co-added in memory after a proper pixel address offset is applied which is based on the traveled distance since the beginning of the full integration time (Mende et al. 2000; Wilkins et al. 2017).

The plasma bubbles are organized along the local magnetic field and good observing conditions from a low inclination limb-viewing satellite can only be achieved if the instrument is pointed along the local magnetic field direction. Therefore a movable turret was added as part of FUV to point the FOV in $10^{\circ}$ steps to up to $\pm30^{\circ}$ from the nominal view direction (Mende et al. 2017). The ICON orbit is predictable and for each nighttime portion of any orbit the local magnetic field direction can be calculated using the IGRF model (Alken et al. 2021). The angle between the spacecraft velocity vector and the magnetic field direction is determined and absolute time sequence commands are generated that move the turret so that it will point within $\pm5^{\circ}$ to that field direction. Knowing the observation geometry, the observations can be mapped to the nominal emission altitude of 300 km in the Spacecraft Orbit-Aligned Position (SOAP) latitude-longitude coordinate space with $8\times8~\text{km}^{2}$ pixel size (Wilkins et al. 2017). Then the TDI technique can be applied to the observations while applying the known horizontal offset to the 100 frames. The result gives the 12 second final integration time. Details are described in more detail in the accompanying paper (Mende et al. 2022).

## Radiometric Calibration: Theory

The FUV instrument was calibrated in a vacuum chamber by illuminating it with monochromatic UV light, measuring the incoming flux, and determining the response of the instrument (Mende et al. 2017). One of the most important properties of the instrument is its passband and its response to photons of different wavelengths (Frey et al. 2017). The careful characterization of the pass band allows then for an on-orbit confirmation or improvement of the calibration accuracy by using regular observations of bright early type-B UV stars (Frey et al. 2003). Furthermore, observing the same stars over the course of the mission allows for the monitoring of the temporal stability of the instrument response.

A star is a point source in any wide-field UV instrument and the response $\text{C}_{\mathrm{s}}$ to incoming star photons in counts/sec can be calibrated against the incoming photon flux $\text{P}_{\mathrm{s}}$ in $\text{photons/cm}^{2}\text{/sec}$ at the entrance aperture as

1 $$ \text{C}_{\mathrm{s}} = \text{P}_{\mathrm{s}} * \varepsilon * \text{A}\quad \bigl[\text{counts/s} = \text{ph/cm}^{2}\text{/s} * \text{counts/ph} * \text{cm}^{2}\bigr] $$

where $\varepsilon $ is the overall efficiency of the system in counts per incoming photon and A is the aperture size in square centimeters. During airglow measurements we observe an extended object that emits $\text{R}_{\mathrm{a}}$ Rayleigh and the instrument responds with a count rate per pixel of $\text{C}_{\mathrm{a}}$. One Rayleigh is defined as an apparent emission rate (radiance) of $10^{6}~\text{photons/cm}^{2}\text{/s/}4\pi\text{sr}$ (Chamberlain 1961). The signal from the airglow is then

2 $$ \text{C}_{\mathrm{a}} = 10^{6}/4\pi * \text{R}_{\mathrm{a}} * \theta * \varepsilon * \text{A}\quad \bigl[\text{counts/s} = \text{ph/cm}^{2}\text{/s/sr/R} * \text{R} * \text{counts/ph} * \text{sr} * \text{cm}^{2} \bigr] $$

where $\theta $ is the solid angle per pixel. For small solid angles $\theta $, the product $\theta *\text{A}$ is the etendu of the system. Replacing $\varepsilon *\text{A}$ with the terms in Eq. (1) gives finally the calibration

3 $$ \text{C}_{\mathrm{a}} = 10^{6}/4\pi * \text{R}_{\mathrm{a}} * \theta * \text{C}_{\mathrm{s}}/\text{P}_{\mathrm{s}} \quad [\text{counts/s}] $$

4 $$ \text{R}_{\mathrm{a}} = \text{C}_{\mathrm{a}} * 4\pi * \text{P}_{\mathrm{s}}/\bigl(10^{6} * \theta * \text{C}_{\mathrm{s}}\bigr)\quad \bigl[\text{R} = \text{counts/s} * \text{cm}^{2} * \text{s} * \text{sr} * \text{R} * \text{ph/} \bigl(\text{ph} * \text{sr} * \text{counts} * \text{cm}^{2}\bigr) \bigr] $$

The number $\text{R}_{\mathrm{a}}$ of Rayleigh emitted by the airglow is then determined from the pixel counts $\text{R}_{\mathrm{a}}$ using $\text{C}_{\mathrm{s}}/\text{P}_{\mathrm{s}}$ with the solid angle $\theta = 8.567\text{E}{-}5$ sr for the FUV science pixel.

## Conversion of Detector Counts to Radiance and Long-Term Stability Monitoring

**Fig. 3:**
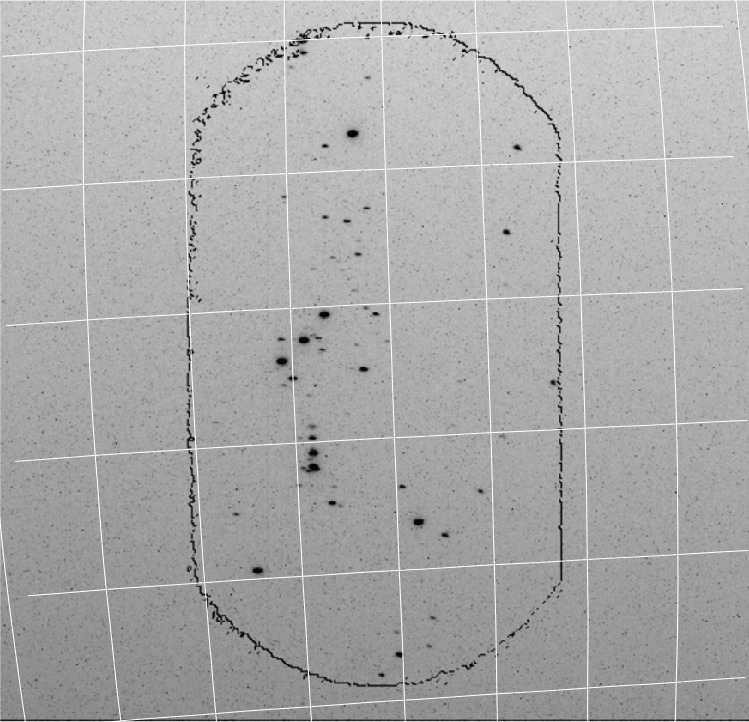
Star calibration image of the LW channel while pointed to the constellation Orion on November 17, 2020. The black contour outlines the border of the imaging area as determined by the shape of the grating. The white lines symbolize the right ascension and declination around the observation region

**Fig. 4:**
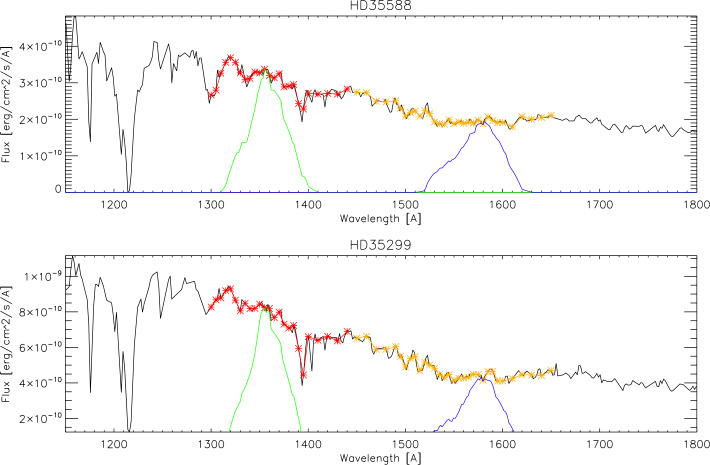
Representative IUE spectra of two stars (HD35588 and HD35299, black lines) together with the wavelength regions (red and orange) that were convolved with the pre-flight measured spectral pass bands of the SW and LW channels (green and blue). The integrals under the green/blue curves represent the portion of the star photons that reach the detectors

Once every month the ICON spacecraft is rotated away from the nominal limb-pointing view of the remote sensing instruments and FUV is pointed towards a pre-determined star field with a large number of bright UV stars (Fig. 3). These star targets were selected before the ICON launch for optimal viewing conditions inside the Earth’s umbra pointing within $45^{\circ}$ around the anti-sunward direction. The quaternions of the orbit and the spacecraft attitude are then used to calculate the view of the instrument and identify individual stars. A catalog of the 1000 brightest UV stars from observations by the International Ultraviolet Explorer IUE (Hartline 1979) is used to determine the response of the FUV instrument by convolving the instrument pass bands with the calibrated IUE spectra (Fig. 4).

**Fig. 5:**
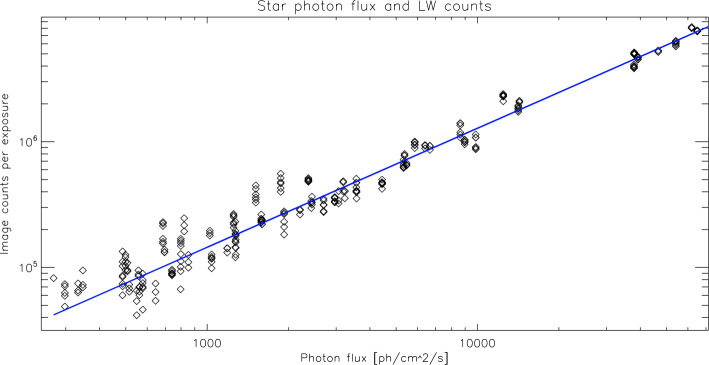
Evaluation of the star observations on 2021-12-26 for the LW channel. The total counts in the 297 individual star observations are plotted over the photon flux from those stars going through the FUV passband. The least squares fit is used to determine the relationship of instrument counts per incoming flux. The correlation coefficient is 0.985

Two different star fields are observed during each star pointing period. Three images are taken each time by both channels. The total instrument counts for the individual stars are then compared to the photon flux going through the instrument to the detector (Fig. 4) and the result for one particular day is shown in Fig. 5. The data show the response of FUV to input photon fluxes of different magnitudes and confirms the ground characterization (Mende et al. 2017). The slope of the fit then provides the conversion from counts per exposure to photon flux from Eq. (4).

**Fig. 6:**
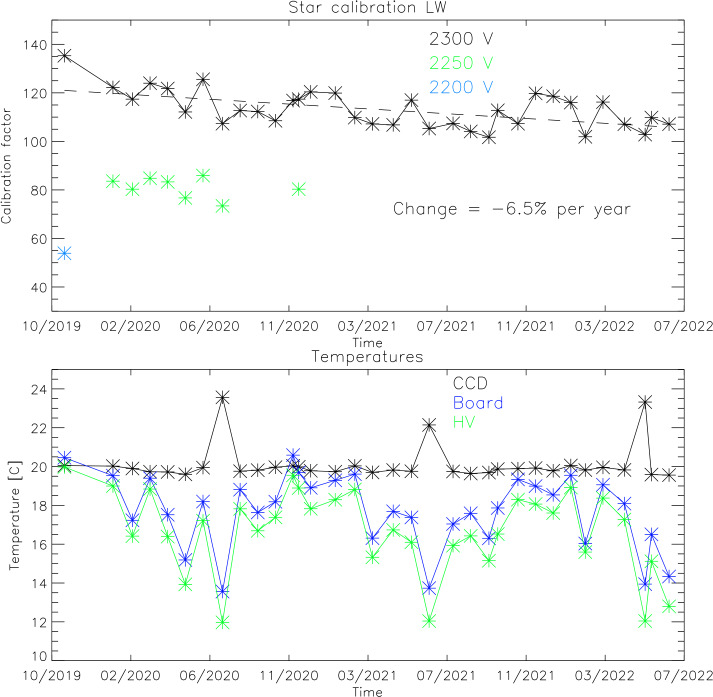

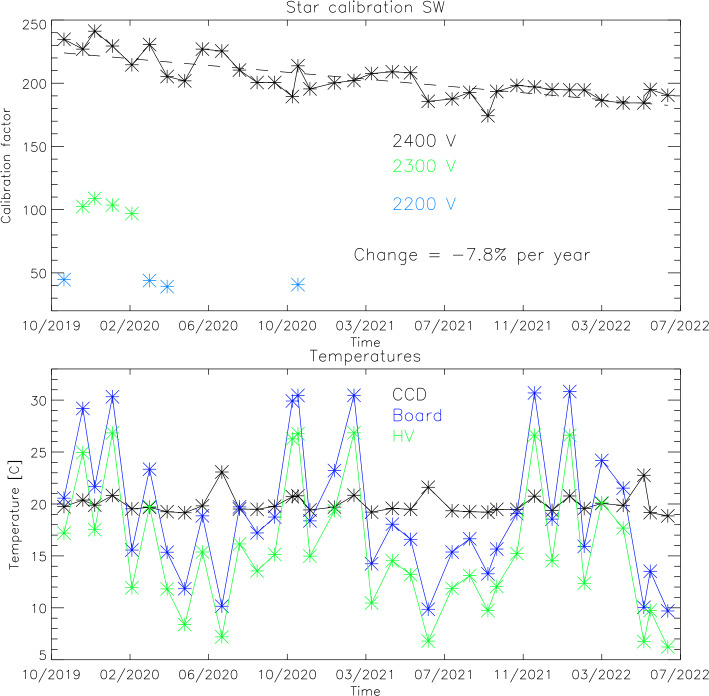
Results of the star calibrations during the first $\sim3$ years of the ICON mission for the LW (top) and SW (bottom) channels. The calibration factors of Counts/s are shown over time as well as temperatures of key components of the detector system that could have an impact on the relative response

All star observations since the beginning of the science mission are used to determine the on-orbit calibration and also to monitor the temporal change of the instrument response. Figure 6 shows the results of this analysis for both channels. Over the $\sim3$ years of the mission the high voltage to the MCPs has been changed two times in order to get a stronger response in terms of counts/Rayleigh and thus improve the signal to noise ratio of the observations. All results in Fig. 6 however are shown as if they were done with the latest high voltage settings of 2300 V for the LW channel and 2400 V for night observations of the SW channel. The relative change of the instrument output for different MCP high voltage settings has been determined during ground calibrations.

The data in the top panel (Fig. 6) show that the relative response of the LW channel has changed (so far) by $-6.5\%$ per year and the third panel shows $-7.8\%$ gain reduction per year for the SW channel. Changes of this order are not unexpected and demonstrate the technical capability of the FUV system to operate in space for many more years. The second and fourth panels of Fig. 6 show the temperatures of key electronics components of the detector system during the star observations, namely the CCD, digital board, and high voltage power supplies (Frey et al. 2017). There are no indications that these temperatures have an impact on the relative response of the detector systems for as long as they stay within the operating range of $5\text{--}30~^{\circ}\text{C}$.

## CCD Background

The FUV instrument uses a combination photocathode-MCP-phosphor-CCD detector system to convert incoming photons into output counts. The background signal of such a system is primarily created by the dark current of the CCD plus much smaller contributions from cosmic ray hits, photon scatter inside the instrument, out-of-band contributions, and bias electrons. The dark current in a semiconductor material is generated because the thermal motion of the silicon atoms tends to thermally excite electrons into the conduction band of the material, which are then finally collected in the individual CCD potential wells (pixels) and counted as signal. The amount of those excited electrons depends on the temperature of the silicon substrate (cooling reduces the dark current) and accumulates linearly with exposure time.

ICON-FUV uses Teledyne Dalsa p/n FTT1010M frame transfer CCD sensors. Those CCDs were selected because of very good experience with similar earlier type detectors on the Imager for Sprites and Upper Atmospheric Lightning (ISUAL) camera (Frey et al. 2016). The CCD has a $1024\times1024$ pixel imaging region and a similar size storage region. Photons are collected for 120 ms in the image section, the collected electrons are shifted into the storage section within 2.3 ms, and the readout takes 116.9 ms. During the 120 ms exposure time and the 2.3 ms frame transfer time, dark current is mostly collected at a constant level over the whole image. However, as the first line of the CCD is read out, all other lines still accumulate dark current in the storage region. This leads to an increasing dark current signal from the bottom of the CCD to the top (Fig. 2, blue line). Generally, the background signal should increase linearly (within statistical fluctuations) from bottom to top, but radiation damaged regions (hot pixels) can lead to increases (for instance at $\text{pixel}=23$ in Fig. 2) which need to be determined and monitored to be properly treated.

**Fig. 7:**
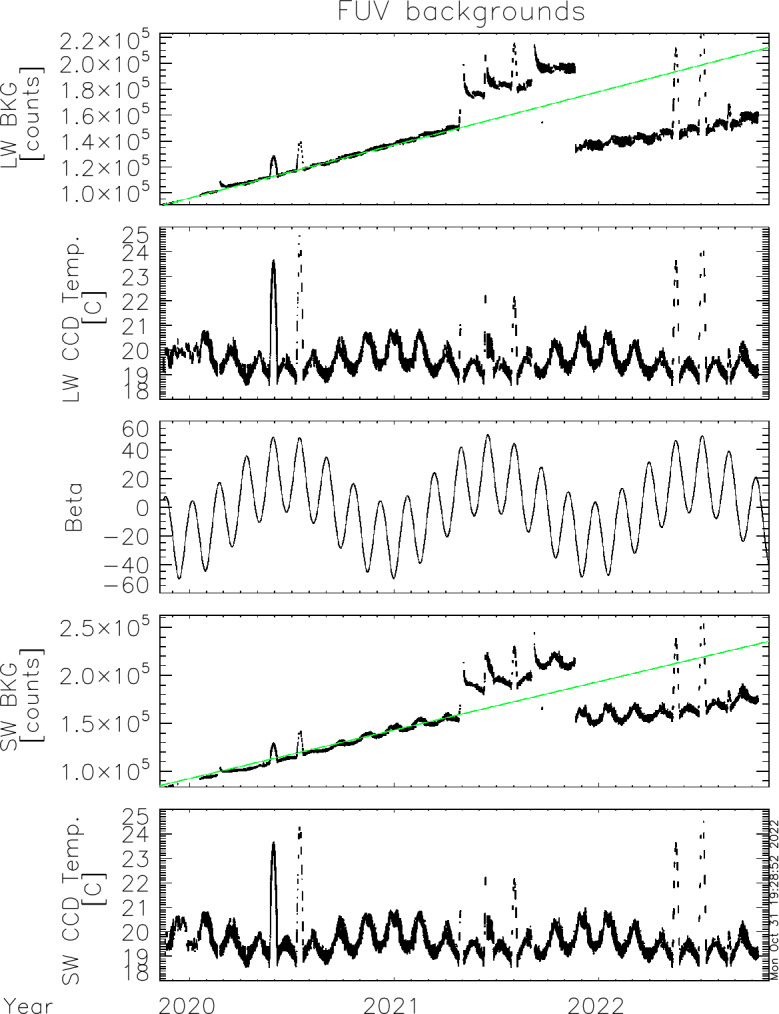
Average background counts in altitude profiles for LW (top) and SW (4th panel) since the beginning of the ICON science mission. The middle panel shows the ICON orbit beta angle and the panels 2 and 5 show the CCD temperatures of the two channels. The green lines in the background plots show the fitted trend from the first 1.5 years of the mission

The ICON satellite regularly flies through the South Atlantic Anomaly (see e.g. Vernov et al. 1967) where the increased flux of energetic particles leads to increased signal fluctuations in all ICON science instruments. A data collection exclusion zone was defined where all remote sensing instruments turn off science data collection and set any high voltages to safe levels in order to prevent electric discharges. Without high voltage to the MCPs the FUV instrument still collects science data and allows monitoring the dark current performance of the CCD. Figure 7 shows the temporal change in the average background counts during the ICON science mission.

There is a general increase of the background signal with time with several strong excursions. Very strong excursions in summer of 2020 and 2021 coincide with peaks of the ICON orbit beta angle. The changed geometry during high beta angle puts sunlight onto radiator panels. The cooling of the detectors is not as efficient as under low-beta conditions and the increased CCD temperature leads to an increase in the dark current and background signal. Very abrupt changes of the background signal on 2021-04-29, 2021-06-13, and 2021-09-02 occurred after anomalies in the spacecraft star trackers which put the observatory into contingency mode. The reason why and how this affected the FUV background levels is still an unsolved issue. In order to slow the background signal increase, the CCD bias was changed on November 20, 2021, which resulted in the strong decrease of the background dark current signal. Further background signal changes will be closely monitored and the bias level potentially changed again to put it at roughly the level from the beginning of the mission.

The instrument background is determined daily and monitored for each integration with the collection of a column of pixels outside of the illuminated CCD chip. This “dynamic background determination” allows for a reliable background determination and subtraction before the signal is transformed into physical units for the science signal.

## Flat Field Determination and Correction

During preflight calibrations, FUV could not be homogeneously illuminated over the entire field of view. So, many point measurements were performed in order to simulate the response to a smooth flat field. On orbit operations permit a much better flat field measurement. Once a month the ICON spacecraft is rotated away from the nominal limb pointing attitude to point the FUV instrument optic axis directly to local nadir. Around the subsolar point the daytime thermosphere is sufficiently homogeneous and evenly illuminated by the solar EUV that the observed scene can be considered a “flat” surface without structure (Frey et al. 2017). Such observations allow the determination of the flat field response of the instrument.

**Fig. 8:**
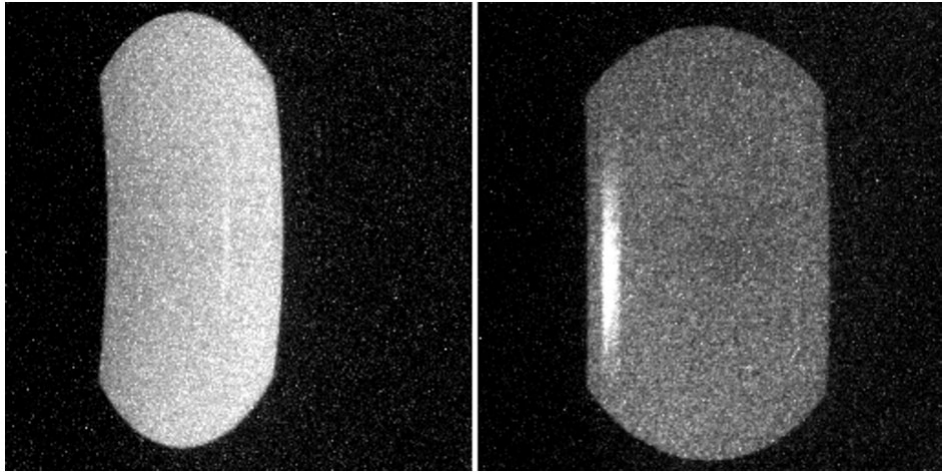
Nadir observations made in engineering mode on 2020-12-11. In engineering mode true integrated 2-dimensional images are collected and shown here after background subtraction. In nadir observations both channels show a “ghost” signal in the right part of the SW image (shown left), and a much stronger “ghost” in the left part of the LW image (shown right)

The FUV nadir observations consist of two parts, science and engineering mode. Two-dimensional images are collected in engineering mode to obtain the true flat field response of the instrument. These nadir observations revealed one unexpected result that both channels show a “ghost” signal (Fig. 8). The most likely cause is a structural element (mirror mount) which reflects photons through the exit slits into the back end cameras. The “ghost” in the SW image is at its maximum 15% brighter than the surrounding area. The “ghost” in the LW channel is at its maximum 130% brighter than the surrounding area. Because the LW channel contains an additional fold mirror (see Fig. 9 of Mende et al. 2017), the ghost appears on the opposite side of the LW channel compared to the SW channel. Other than these “ghosts” the responses of both channels appear reasonably flat in the vertical direction ($<5\%$). The SW channel (left in Fig. 8) shows a left-right variation which is caused by the geometric imaging properties of that channel (see Sect. 3 and Fig. 1). This deviation is corrected during the science data processing.

**Fig. 9:**
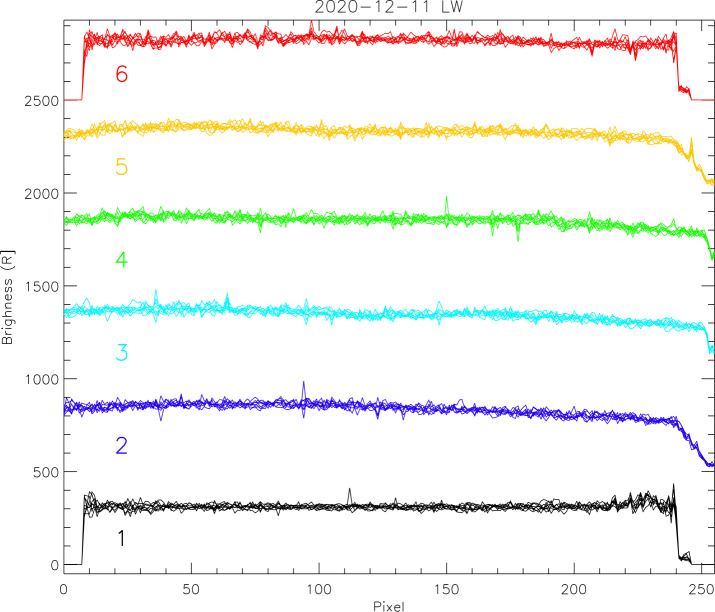
Airglow brightness profiles collected in science mode during nadir view of the instrument on 2020-12-11 by the LW channel. The profiles of eight consecutive data collections (each 12 seconds apart) are plotted on top of each other and the colors are for the six different profiles (numbers in Fig. 1). The profiles are offset by 500 R from each other for better visibility

The instrument is also operated in science mode when the co-adding of the raw pixels generates science profiles in the same way as during limb pointing (see Sect. 3, Altitude Profile Data Collection). These profiles show the airglow brightness along the spacecraft track during nadir view. The results of one particular day are given in Fig. 9 for the LW channel. The profiles plotted in black are for the leftmost region of the LW image where the “ghost” would show up as a strong increase in signal brightness. The flat field correction during science data processing removes this effect and the final profiles are flat. Some profiles show signal increases that were generated by energetic charged particles (protons) or cosmic rays hitting the CCD detector during the integration time. These signal increases are short lived and not real “hot” pixels which would be permanently damaged. The co-adding of the altitude profiles reduces the relative impact any real “hot” pixel would have.

## Altitude Determination

**Fig. 10:**
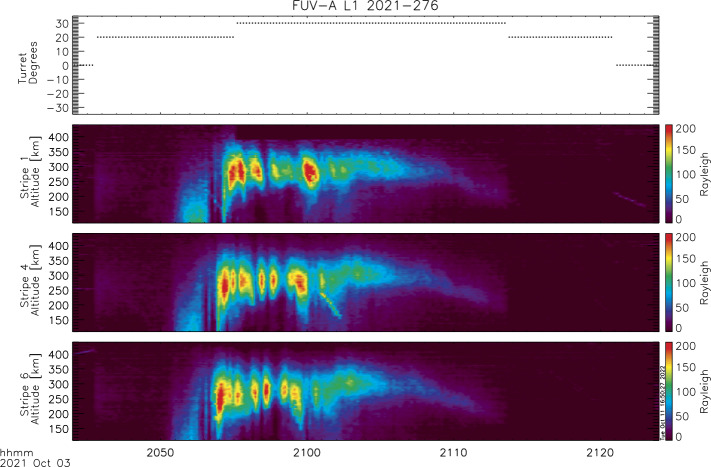
A nightside observation of the SW channel showing brightness modulations due to plasma depletions on October 3, 2021 between 20:45 to 21:25. The turret angle (top panel) was changed during that observation to provide the best viewing geometry. Stripe numbers correspond to the labels in Fig. 1-F. Stripe 1 averages the observations between $9\text{--}6^{\circ}$ looking backwards from the center view direction through the turret. Stripe 4 averages the observations between the center direction at $0^{\circ}$ and $3^{\circ}$ forward. Stripe 6 looks forward $6\text{--}9^{\circ}$ from the center view and sees airglow structures first

Generally the spacecraft orbit and attitude quaternions should allow for the exact calculation of the view direction and tangent altitude for each pixel. This is certainly true for the two-dimensional viewing of FUV like in the star image in Fig. 3. However, the remapping of raw pixels into science pixels requires high accuracy determination of the attitude and the pointing knowledge of pixels had to be verified on orbit. Stars were used for this verification. As the individual altitude profiles are $3^{\circ}$ wide accumulations, a star can provide the exact vertical and horizontal viewing angles only at the moment when it moves from one profile into the next. The vertical viewing angle can be determined with an accuracy equivalent to 1/2 of the pixel size. A number of stars appearing at different elevation angles in the limb altitude profile of the science data provided a relationship between pixel number and tangent altitude. An example of the conversion to altitude space (ordinate) is shown in Fig. 10, which shows images of nighttime plasma depletions (data level-1) for one particular orbit at night. The instrument turret was moved to $+20^{\circ}$ and $+30^{\circ}$ for optimal viewing along the direction of the local magnetic field. The time series of three profiles (1, 4, and 6; see Fig. 1) are shown in calibrated brightness and altitude above the surface of the Earth. A clear modulation of the signal can be seen that is caused by plasma density depletions.

## Two-Dimensional TDI Imaging of the Nighttime Ionosphere

**Fig. 11:**
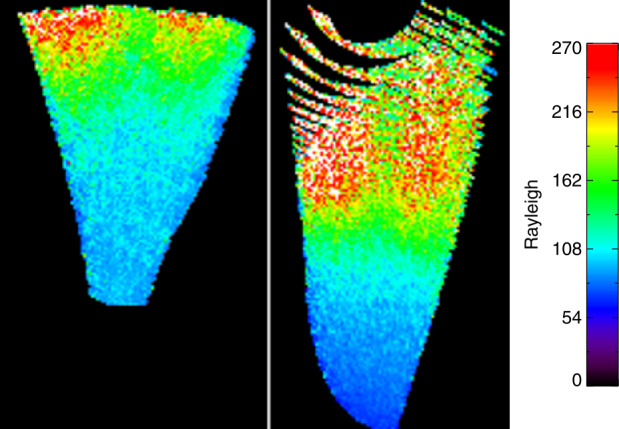
One record of 2-dimensional TDI images with the limb portion at the left and the sublimb portion at the right. The raw data were mapped on-board to 300 km altitude with a pixel resolution of $8\times8~\text{km}^{2}$

The TDI process and mapping of images as described in Sect. 4 have been used to map the nightside airglow observations onto the globe and investigate the structure and variability of the nighttime ionosphere. The observations are mapped into two parts as limb and sublimb observations. Pixels in limb observations of tangent heights greater than 300 km are mapped to the geographic location of the tangent point of the rays associated with the pixels. Pixels in sub-limb observations are mapped to the point where the associated rays cross the 300 km altitude region (see details in Wilkins et al. 2017; Mende et al. 2022). A single 12 second exposure record is given in Fig. 11 where a clear depletion can be seen, especially in the sub-limb part center.

**Fig. 12:**
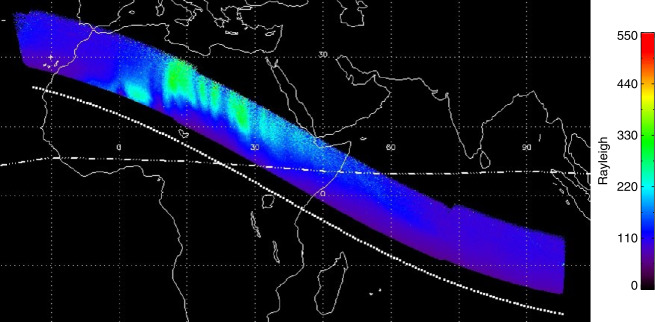
Example of the mapped sublimb observations during a nightside portion of an orbit on the 9th of October 2021. The dotted line shows the geographic position of the ICON spacecraft. The dash-dotted line is the magnetic equator. The sublimb observations show the airglow band north of the magnetic equator with indications of field-aligned plasma depletions. These observations cover the same time as the altitude profiles shown in Fig. 10

Knowing the speed of the spacecraft and the pointing of FUV, maps of the airglow distribution can be constructed by co-adding images like those shown in Fig. 11 and spatially shifting them to follow the ICON spacecraft motion on orbit for each nightside orbit portion. An example of such an ionospheric map is given on Fig. 12, which shows the spatial distribution of the northern Equatorial Ionospheric Anomaly (EIA) at $\sim15^{\circ}$ magnetic north. There are magnetic field aligned plasma depletions or Equatorial Plasma Bubbles. More details are described in the accompanying paper (Mende et al. 2022 and references therein).

## Science Results

**Fig. 13:**
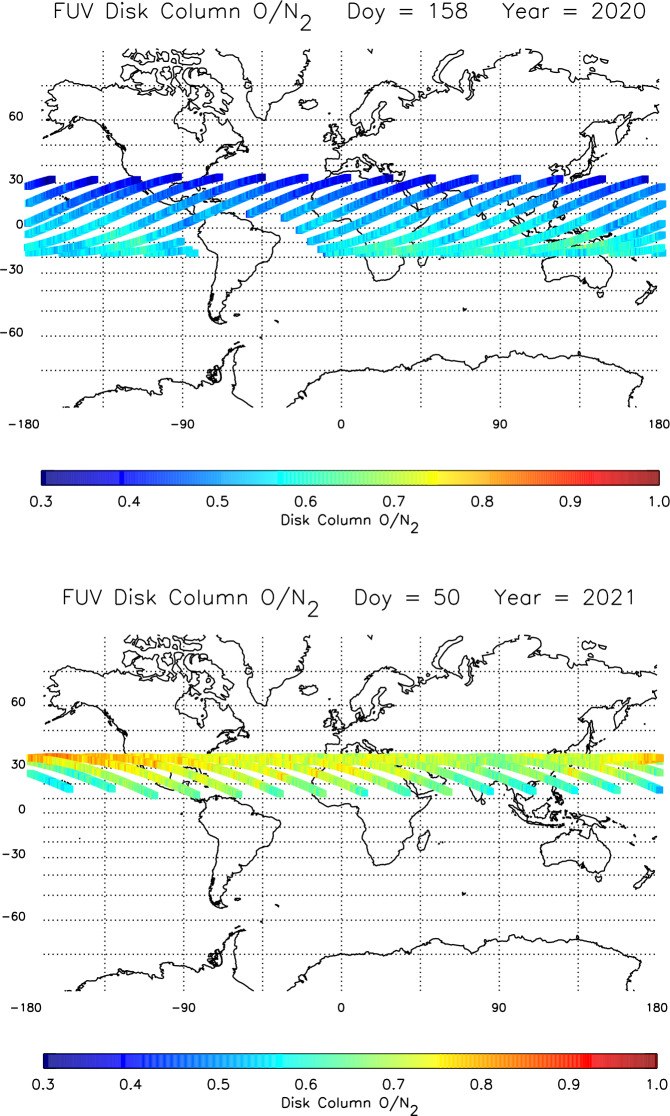
Seasonal variation of the thermospheric column O/N_2_ ratio derived from ICON FUV disk viewing data measured on June 6, 2020 (upper panel) and February 19, 2021 (lower panel). Each panel illustrates data from a single day, color coded along the ICON orbit at the location where the FUV instrument line-of-sight passes through 150 km altitude. No data are taken inside the South Atlantic Anomaly. The solar 10.7 cm flux was 73.8 for the upper panel and 70.5 for the lower panel

As already mentioned in the introduction, the two measurement objectives for the FUV instrument are the determination and monitoring of the dayside thermospheric composition, and of the nightside oxygen ion density. The separate and simultaneous measurements of the SW and LW channels are used to determine the altitude profiles of daytime atomic oxygen and molecular nitrogen and determine the thermospheric column O/N_2_ ratio (Stephan et al. 2018; Meier 2021). An example of the seasonal variability of the column O/N_2_ is shown in Fig. 13. The map in the upper panel illustrates northern summer solstice conditions when the ratio decreases towards the higher northern latitudes. The opposite behavior is seen in the lower panel map of northern winter conditions. This seasonal variability is completely consistent with TIMED/GUVI observations (albeit at much higher solar activity) as well as the NRLMSIS00 empirical model and first-principles global circulation models (Strickland et al. 2004).

ICON column O/N_2_ data have been used to determine the impact of tides on thermospheric composition (England et al. 2021). Combining data from several ICON instruments along with a global circulation model, it was demonstrated, that during morning hours and at latitudes away from the peak of the equatorial ionospheric anomaly, the impact of nonmigrating tides on thermospheric composition can be observed, even though it is not as big as might be expected.

In addition to the FUV instrument, ICON also carries the Extreme Ultraviolet instrument (EUV) for the determination of the ionospheric $\text{O}^{+}$ density from dayglow measurements between 54–88 nm (Sirk et al. 2017). Besides the standard $\text{O}^{+}$ ion emissions at 61.6 nm and 83.4 nm EUV also observes other emissions that can be used to determine the neutral atmosphere (Tuminello et al. 2022). A nitrogen molecule feature at 87.8 nm opens the path to determine the column O/N_2_ ratio from EUV measurements. The comparison between EUV and FUV measurements provided a good correlation between the results, but further work is needed on the inversion of EUV measurements (Tuminello et al. 2022).

Nightside observations of FUV enabled the determination of the F-region ionospheric oxygen ion density (effectively equal to the electron density) following the method described in Kamalabadi et al. (2018). A comparison of the ion peak height hmF2 and peak density NmF2 with measurements by the COSMIC-2 constellation (Cook et al. 2013) and ionosondes revealed that the FUV observations are consistent with the COSMIC-2 and ionosonde measurements, with an average density bias lower than $1 \times 10^{11}~e\text{/m}^{3}$. When restricting the analysis to cases having an NmF2 value larger than $5\times10^{11}~e\text{/m}^{3}$, FUV provides the peak electron density with a mean difference with COSMIC-2 of 10% (Wautelet et al. 2021). The peak altitude, also determined from FUV observations, is found to be 15 km above that obtained from COSMIC-2, and 38 km above the ionosonde value on average.

**Fig. 14:**
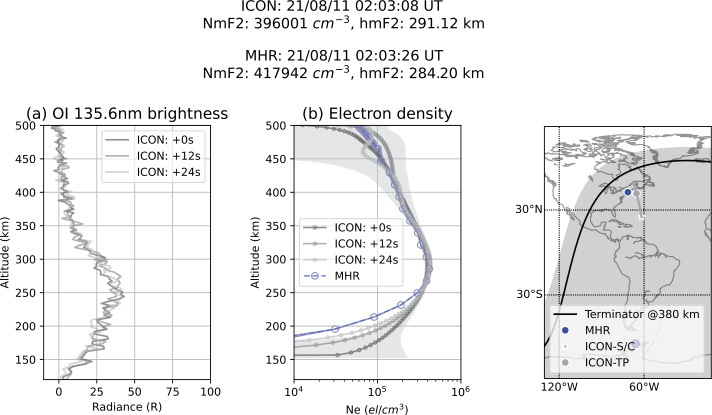
Comparison between nightside measurements of ICON-FUV and the Millstone Hill Incoherent Scatter Radar (MHO). The derived ionospheric peak densities agree within 10% and the peak altitude difference is within the radar altitude resolution

Another confirmation of the FUV data quality was obtained through the comparison to radar measurements by the Millstone Hill Incoherent Scatter Radar Observatory (MHO). The probed volume of the radar is regularly within the region observed by ICON-FUV. During 38 coordinated radar measurements in 2021 the results were compared. An example is given in Fig. 14, which shows the measured FUV brightness and the estimated $\text{O}^{+}$ density by ICON (Kamalabadi et al. 2018) together with the measured electron density by MHO and the associated airglow brightness. The NmF2 results of both measurements agree within 10% and the hmF2 difference is within the altitude resolution of the radar of 18 km. The FUV and MHO measurements are in good agreement within the error bars from 250 km up to 400 km in altitude. Below 250 km the electron density observed by ICON is overestimated due to the fact that a simplified isothermal atmosphere was considered when estimating the electron density. The lower the altitude, the higher the overestimation (Qin et al. 2015).

Photoionization of the neutral atmospheric species by solar UV radiation and X-rays produces energetic electrons in the ionosphere, also known as photoelectrons. Conjugate photoelectrons (CPEs) refer to those PEs that travel along the magnetic field lines from one hemisphere to the other and lose their energy through collisions with neutral particles, ambient plasma, and via wave-particle interactions in the plasmasphere. As the CPEs descend into the conjugate atmosphere, many retain sufficient energy ($>10~\text{eV}$) to produce collisionally excited O atoms that later emit 135.6 nm photons. Emission from CME production must be removed from the radiative recombination signal used to routinely observe the ionosphere at night. By combining global scale far-UV measurements by ICON and radio-occultation measurements from COSMIC2 a method was developed to estimate the incident photoelectron’s energy spectra as a function of altitude (Urco et al. 2021). Quantification of photoelectron impact is enabled by the fact that CPEs directly affect FUV airglow emissions but not radio occultation measurements. The authors showed that a significant fraction of ICON-FUV measurements is affected by CPEs during the winter solstice. A comparison of estimated photoelectron fluxes with measured photoelectron spectra was used to gain further insights into the estimation method and produces consistent values within the 10–60 eV PE energy range.

In a recent comparison of FUV measurements of ionospheric density profiles with radio occultation measurements by Cosmic-2 and ionosonde measurements from the ground, the results of Wautelet et al. (2021) were expanded and substantially enhanced (Wautelet et al. 2023). ICON FUV data between December 2019 and August 2022 with improved calibration and background subtraction, better star removal, and stronger data quality requirements, allowed for a direct comparison between NmF2 and hmF2 determinations by the three measurement methods. The study found that the FUV determined peak density and height are, on average, similar to radio-based observations by 6–11% in density and 7 km in height. This confirms that ICON-FUV provides peak characteristics compatible with established ionospheric datasets based on radio signals. The authors conclude that FUV reliably monitors the peak density and height with an accuracy compatible to that of external data sources (Wautelet et al. 2023).

## Conclusions

The Far Ultraviolet (FUV) instrument on the ICON spacecraft has been collecting science data for about 3 years since the official beginning of the science mission in November 2019. Regular star observations are used to determine the response of the instrument to known photon flux from bright UV stars. Monitoring over the time of mission revealed a 6–8% decrease in response by both channels, which is well within the expected range. The derived response values are then used to calculate the airglow brightness and derive science parameters about the composition and density of the ionosphere. Comparisons with measurements by other instruments (COSMIC-2, ionosondes, and incoherent radar) show generally a good agreement within the measurement uncertainties. Accompanying papers in this issue describe obtained science results in more detail.
